# Supplementary material for: Heat adaptation of phage T7 under an extended genetic code
Source: Virus Evol. 2021 Dec 1;7(2):veab100. doi: 10.1093/ve/veab100 (PMC8923235; doi:10.1093/ve/veab100)
Supplement: veab100_Supp [file veab100_supp.zip › Sequencing_Analysis.zip!/Sequencing_Analysis/A2/evidence/RA_35.html]

BRESEQ :: Evidence


| Read alignment evidence... | | | | | | | | | | | |
| --- | --- | --- | --- | --- | --- | --- | --- | --- | --- | --- | --- |
|  | seq id | position | | ref | new | freq | score (cons/poly) | reads | annotation | genes | product |
| \* | NC\_001604.1 | 35,266 | 0 | A | G | 41.1% | 126.3 / inf | 448 | intergenic (–/–) | *–/–* | –/– |
| Reads supporting (aligned to +/- strand):  **ref** base A (153/111);  **new** base G (97/87);  **total** (250/198) | | | | | | | | | | | |
| Fisher's exact test for biased strand distribution *p*-value = 2.88e-01 | | | | | | | | | | | |
| Kolmogorov-Smirnov test that lower quality scores support variant *p*-value = 7.31e-01 | | | | | | | | | | | |

Only 100 of 458 total aligned reads displayed.  

`ACCTCACTACGGATACTATCGGTGTCAATAACGATGGTCACTTGGATGCTCGTGGTCGTCGAATTGTGAACCTAGCGAACGCCGTGGATGACCGCGATGCTGTTCCGTTTGGTCAACTAAAGACCATGAACCAGAACTCATGGCAAGCACGTAATGAAGCCTTACAGTTCCGTAATGAGGCTGAGACTTTCAGAAACCAAGCGGAGGGCTTTAAGAACGAGTCCAGTACCAACGCTACGAACACAAAGCAGTGGCGCGATGAGACCAAGGGTTTCCGAGACGAAGCCAAGCGGTTCAAGAATACGGCTGGTCAATACGCTACATCTGCTGGGAACTCTGCTTCCGCTGCGCATCAATCTGAGGTAAACGCTGAGAACTCTGCCACAGCATCCGCTAACTCTGCTCATTTGGCAGAACAGCAAGCAGACCGTGCGGAACGTGAGGCAGACAAGCTGGAAAATTACAATGGATTGGCTGGTGCAATTGATAAGGTAGATGGAACCAATGTGTACTGGAAAGGAAATATTCACGCTAACGGGCGCCTTTACATGACCACAAACGGTTTTGACTGTGGCCAGTATCAACAGTTCTTTGGTGGT  >  NC_001604.1/34967‑35565`  

                                                                                                                                                                                                                                                                                                           `|`                                                                                                                                                                                                                                                                                                             
`aCCTCACTGCGGATACTATCGGTGTCAATAACGATGGTCACTTGGATGCTCGTGGTCGTCGAATTGTGAACCTAGCGAACGCCGTGGATGACCGCGATGCTGTTCCGTTTTGTCAACTAAAGACCATGAACCAGAACTCATGGCAAGCACGTAATGAAGCCTTACAGTTCCGTAATGAGGCTGAGACTTTCAGAAACCAAGCGGAGGTCTTTAAGAACGAGTCCAGTACCAACGCTACGAACACAAAGCAGTGGCGCGATGAGACCAAGGGTTTCCGAGACGAAGCCAAGCGGTTCAAGaa                                                                                                                                                                                                                                                                                                            <  1:758701/301‑1 (MQ=255)`  
`aCCTCACTGCGGATACTATCGGTGTCAATAACGATGGTCACTTGGATGCTCGTGGTCGTCGAATTGTGAACCTAGCGAACGCCGTGGATGACCGCGATGCTGTTCCGTTTGGTCAACTAAAGACCATTGACCAGAACTCATGGCAAGCACGTAATGAAGCCTTACAGTTCCGTAATGAGGCTGAGACTTTCAGAAACCAAGCGGAGGGCTTTAAGAACGAGTCCAGTACCAACGCTACGAACACAAAGCAGTGGCGCGATGAGACCAATGGTTTCCGAGACGAAGCCGAGCGGTTCAAGaa                                                                                                                                                                                                                                                                                                            >  1:160935/1‑301 (MQ=255)`  
      `ctgcGGATACTATCGGTGTCAATAACGATGGTCACTTGGATGCTCGTGGTCGTCGAATTGTGAACCTAGCGAACGCCGTGGATGACCGCGATGCTGTTCCGTTTGGTCAACTAAAGACCATGGACCAGAACTCATGGCAAGCACGTAATGAAGCCTTACAGTTCCGTAATGAGGCTGAGACTTTCAGAAACAAAGCGGAGGGCTTTAAGAACGAGTCCAGTACCAACGCTACGAACACAAAGGAGTGGCGCGATGAGACCAAGGGTTTCCGAGACGAAGCCAAGCGGGTCAAGaactgtct                                                                                                                                                                                                                                                                                                      >  1:132918/4‑295 (MQ=255)`  
        `gcGGATACTATCGGTGTCAATAACGATGGTCACTTGGATGCTCGTGGTCGTCGAATTGTGAACCTAGCGAACGCCGTGGATGACCGCGATGCTGTTCCGTTTGGTCAACTAAAGACCATGGACCAGAACTCATGGCAAGCACGTAATGAAGCCTTACAGTTCAGTAATGAGGCTGAGACTTTCAGAAACCAAGCGGAGGGCTTTAAGAACGAGTACAGTACCAACGCTACGAACACAAAGCAGTGGCGCGATGAGACCAAGGGTTTCCGAGACGAAGCCAAGAGGTTCAAGGATACGGcat                                                                                                                                                                                                                                                                                                    >  1:753979/2‑299 (MQ=255)`  
           `tcggagatgtgtataagagacagtGGTCACTTGGATGCTCGTGGTCGTCGAATTGTGAACCTAGCGAACGCCGTGGATGACCGCGATGCTGTTCCGTTTGGTCAACTAAAGACCATGGACCAGAACTCATGGCAAGCACGTAATGAAGCCTTACAGTTCCGTAATGAGGCTGAGACTTTCAGAAACCAAGCGGAGGGCTTTAAGAACGAGTCCAGTACCAACGCTACGAACACAAAGCAGTGGCGCGATGAGACCAAGGGTTTCCGAGACGAAGCAAAGCGGTTCAAGAATACGGCTGGTc                                                                                                                                                                                                                                                                                                 <  1:69953/278‑1 (MQ=255)`  
           `gATACTATCGGTGTCAATAACGATGGTCACTTGGATGCTCGTGGTCGTCGAATTGTGAACCTAGCGAACGCCGTGGATTACCGCGATGCTGTTCCGTTTGGTCAACTAAAGACCATGGACCAGAACTCATGGCAAGCACGTAATGAAGCCTTACAGTTCCGTAATGAGGCTGAGACTTTCAGAAACCAAGCGGAGGGCTTTAAGAACGAGTCCAGTACCAACGCTACGAACACAAAGCAGTGGCGCGATGAGACCAAGGGTTTCCGAGACGAAGCCAAGCGGTTCAAGAATACGGCTGGTc                                                                                                                                                                                                                                                                                                 >  1:413361/1‑301 (MQ=255)`  
             `tACTATCGGTGTCAATAACGATGGTCACTTGGATGCTCGTTGTCGTCGAATTGTGAACCTAGCGAACGCCGTGGATGACCGCGATGCTGTTCCGTTTGGTCAACTAAAGACCATGGACCAGAACTCATGGCAAGCACGTAATGAAGCCTTACAGTTCCGTAATGAGGTTGAGACTTTCAGAAACCAAGCGGAGGGCTTTAAGAACGAGTCCAGTACCAACGCTACGAACACAAAGCAGTGGCGCGATGAGACCAAGGGTTTCCGAGACGAAGCCAAGCGGTTCAAGGATACGGCTGGTCaa                                                                                                                                                                                                                                                                                               >  1:285341/1‑301 (MQ=255)`  
               `cTATTGGTGTCAATAACGATGGTCACTTTGATGCTCGTGGTCGTCGAATTGTGAACCTAGCGAACGCCGTGGATGACCGCGATGCTGTTCCGTTTGGTCAACTAAAGACCATGGACCAGAACTCATGGCAAGCACGTAATGAAGCCTTACAGTTCCGTAATGAGGCTGAGACTTTCAGAAACCAAGCGGAGGGCTTTAAGAACGAGTCCAGTACCAACGCTACGAACACAAAGCAGTGGCGCGATGAGACCAAGGGTTTCCGAGACGAAGCCAAGCGGTTCAAGAATACGGCTGGTCAATa                                                                                                                                                                                                                                                                                             >  1:820352/1‑301 (MQ=255)`  
               `cTATCGGTGTCAATAACGATGGTCACTTGGATGCTCGTGGTCGTCGAATTGTGAACCTAGCGAACGCCGTGGATGACCGCGATGCTGTTCCGTTTGGTCAACTAAAGACCATTGACCAGAACTCATGGCAAGCACGTAATGAAGCCTTACAGTTCCGTAATGAGGCTGAGACTTTCAGAAACCAAGCGGAGGGCTTTAAGAACGAGTCCAGTACCAACGCTACGAACACAAAGCAGTGGCGCGATGATACCAAGGGTTTCCGAGACGAAGCCGAGCGGTTCAAGAATAcctgtctcttata                                                                                                                                                                                                                                                                                             >  1:8660/1‑289 (MQ=255)`  
                `gacaggTGTCAATAACGATGGTCACTTGGATGCTCGTGGTCGTCGAATTGTGAACCTAGCGAACGCCGTGGATGACCGCGATGCTGTTCCGTTTGGTCAACTAAAGACCATGGACCAGAACTCATGGCAAGCACGTAATGAAGCCTTACAGTTCCGTAATGAGGCTGAGACTTTCAGAAACCAAGCGGAGGGCTTTAAGAACGAGTCCAGTACCAACGCTACGAACACAAAGCAGTGGCGCAATGAGACCAAGGGTTTCCGAGACGAAGCCGAGCGGTTCAAGAATACGGCTGGTCAATAc                                                                                                                                                                                                                                                                                            <  1:615301/297‑1 (MQ=255)`  
                `aagagacagcAATAACGATGGTCACTTGGATGCTCGTGGTCGTCGAATTGTGAACCTAGCGAACGCCGTGGATGACCGCGATGCTGTTCCGTTTGGTCAACTAAAGACCATGGACCAGAACTCATGGCAAGCACATAATGAAGCCTTACAGTTCCGTAATGAGGCTGAGACTTTAAGAAACCAAGCGGAGGGCTTTAAGAACGAGTCCAGTACCAACGCTACGAACACAAAGCAGTGGCGCGATGAGACCAAGGGTTTCCGAGACGAAGCCGAGCGGTTCAAGAATACGGCTGGTCAATAc                                                                                                                                                                                                                                                                                            <  1:198100/292‑1 (MQ=255)`  
                     `gtgtCAATAACGATTGTCACTTGGATGCTCGTGGTCGTCGAATTGTTAACCTAGCTAACGCCGTGGATGACCGCGATGCTGTTCCGTTTGGTCAACTAAAGACCATGGACCAGAACTCATTGCAAGCACGTAATGAAGCCTTACAGTTCCGTAATGAGGCTGAGACTTTCAGAAACCAAGCTGAGGGCTTTAAGAACGAGTCCAGTACCAACGCTACGAACACAAAGCAGTGGCGCGATGAGACCAAGGGTTTCCGAGACGAAGCCGAGCGGTCCAAGAATACGGCTGGTCAATAcctttt                                                                                                                                                                                                                                                                                       >  1:761465/1‑296 (MQ=255)`  
                     `gtgtCAATAACGATGGTCACTTGGATGCTCGTGGTCGTCTAATTGTGAACCTAGCGAACGCCGTGGATGACCGCGATGCTGTTCCGTTTGGTCAACTAAAGACCATGGACCAGAACTCATGGCAAGCACGTAATGAAGCCTTACAGTTCCGTAATGAGGCTGAGACTTTCAGAAACCAAGCGGAGGGCTTTAAGAACGAGTCCAGTACCAACGCTACGAACACAAAGCAGTGGCGCGATGAGACCAAGGGTTTCCGAGACGAAGCCAAGCGGTTCAAGGATACGGCTGGTCAATACGCTAc                                                                                                                                                                                                                                                                                       <  1:495941/301‑1 (MQ=255)`  
                       `gtCAATAACGATGGTCACTTGGATGCTCGTGGTCGTCGAATTGTGAACTTAGCGAACGACGTCGATGAACGCGATGCTGTTCCGTTTGGTCAACTAAAGATCATGGACCAGAACTCATGGCTAGCACGTAATGAAGACTTACAGTTCCGTAACGAGGTTGCGACTTTCAGAAAACAAGCAGAGGGCTTAAAGAACGAGTCCAGTACTTACGCTACGAACATAAAGGAGTGGCGCTATGAGAACAAGGGTTTCCGAAACGAAGCAGATCGGTTCAAGAATACGTCTGGTCAGTActgatcct                                                                                                                                                                                                                                                                                     >  1:343743/1‑294 (MQ=255)`  
                       `gtCAATAACGATGGTCACTTGGATGCTCGTGGTCGTCGAATTGTGAACCTAGCGAACGCCGTGGATGACCGCGATGCTGTTCCGTTTGGTCAACTAAAGACCATGGACCAGAACTCATGGCAAGCACGTAATGAAGCCTTACAGTTCAGTAATGAGGCTGAGACTTTCAGAAACCAAGCGGAGGGCTTTAAGAACGAGTCCAGTACCAACGCTACGAACACAAAGCAGTGGCGCGATGAGACCAAGGGTTTCCGAGACGAAGCCGAGCGGTTCAAGAATACGGCTGGTcctgtctcttata                                                                                                                                                                                                                                                                                     >  1:684794/1‑289 (MQ=255)`  
                         `cAATAACGATGGTCACTTGGATGCTCGTGTTCGTCGAATTGTGAACCTAGCGAACGCCGTGGATGACCTCGATGCTGTTCCGTTTTGTCAACTAAAGACCATGGACCAGAACTCATGGCAAGCACGTAATGAAGCCTTACAGTTCCGTAATTAGGCTGAGACTTTCAGAAACCAAGCTGAGGGCTTTAAGAACTATTCCAGTACCAACGCTACGAACACAAATCAGTGGCGCGATTAGACCAAGGGTTTCCGAGACGAAGCCAAGCGGTTCAAGGATACGGCTGGTCAATACGCTACATct                                                                                                                                                                                                                                                                                   >  1:94753/1‑301 (MQ=255)`  
                                `gATGGTCACTTGGATGCTCGTGGTCGTCGAATTGTGAACCTAGCGAACGCCGTGGATGACCGCGATGCTGTTCCGTTTGGTCAACTAAAGACCATGGACCAGAACTCATGGCAAGCACGTAATGAAGCCTTACAGTTCCGTAATTAGGCTGAGACTTTCAGAAACCAAGCGGAGGGCTTTAAGAACGAGTCCAGTACCAACGCTACGAACACAAAGCAGTGGCGCGATGAGACCAAGGGTTTCCGAGACGAAGCCAAGCGGTTCAAGGATACGGCTGGTCAATACGCTACATCTGCTGGGa                                                                                                                                                                                                                                                                            <  1:524147/301‑1 (MQ=255)`  
                                           `ggATGCTCGTGGTCGTCGAATTGTGAACCTAGCGAACGCCGTGGATGACCGCGATGCTGTTCCGTTTGGTCAACTAAAGACCATGGACCAGAACTCATGGCAAGCACGTAATGAAGCCTTACAGTTCCGTAATGAGGCTGAGACTTTCAGAAACCAAGCGGAGGGCTTTAAGAACGAGTCCAGTACCAACGCTACGAACACAAAGCAGTGGCGCGATGAGACCAAGGGTTTCCGAGACGAAGCCAAGCGGTTCAAGGATACGGCTCGTCAATACGCTACATcctgtctcttatacacatct                                                                                                                                                                                                                                                                 >  1:174804/1‑282 (MQ=255)`  
                                             `aTGCTCGTGGTCGTCGAATTGTGAACCTAGCGAACGCCGTGGATGACCGCGATGCTGTTACGTTTGGTCAACTAAAGACCATGGACCAGAACTCATGGCAAGCACGTAATGAAGCCTTACAGTTCCGTAATGAGGATGAGACTTTCAGAAACCAAGCGGAGGGCTTTAAGAACGAGTCCAGTACCAACGCTACGAACACAAAGCAGTGGCGCGATGAGACCAAGGGTTTCCGAGACGAAGCCAAGCGGTTCAAGGATACGGCTGGTCAATACGCTACATCTGCTGGGAACTCTGCTTCCGc                                                                                                                                                                                                                                                               <  1:791043/301‑1 (MQ=255)`  
                                                       `tcgtcgAATTGTGAACCTAGCGAACGCCGTGGATGACCGCGATGCTGTTCCGTTTGGTCAACTAAAGACCATGGACCAGAACTCATGGCAAGCACGTAATGAAGCCTTACAGTTCCGTAATGAGGCTGAGACTTTCAGAAACCAAGCGGAGGGCTTTAAGAACTAGTCCAGTACCAACGCTACGAACACAAAGCAGTGGCGCGATGAGACCAAGGGTTTCCGAGACGAAGCCGAGCGGTTCAAGAATACGGCTGGTCAATACGCTACATCTGCTCGGAACTCTGCTTCCGCTGCGCATCaa                                                                                                                                                                                                                                                     <  1:114160/301‑1 (MQ=255)`  
                                                         `gtcgAATTGTGAACCTAGCGAACGCCGTGGATGACCGCGATGCTGTTCCGTTTGGTCAACTAAAGACCATGGACCAGAACTCATGGCAAGCACGTAATGAAGCCTTACAGTTCCGTAATGAGGCTGAGACTTTCAGAAACCAAGCGTAGGGCTTTAAGAACGAGTCCAGTACCAACGCTACGAACACAAAGCAGTGGCGCGATGAGACCAATGGTTTCCTAGACGAAGCCGAGCGTTTCAAGAATACGGCTGGTCAATACGCTACATCTGCTGGGAACTCTGCTTCCGCGTCGCCTCAATc                                                                                                                                                                                                                                                   >  1:101536/1‑301 (MQ=255)`  
                                                              `aTTGTGAACCTAGCGAACGCCGTGGATGACCGCGATGCTGTTCCGTTTGGTCAACTAAAGACCATGGACCAGAACTCATTGCAAGCACGTAATGAAGCCTTACAGTTCCGTAAGGAGGCTGAGACTTTCAGAAACCAAGCGTAGGGCTTTAAGAACGAGTCCAGTACCAACGCTACGAACACAAAGCAGTGGCGCGATGAGACCAAGGGTTTCCTAGACGAAGCCAAGCGGTTCAAGAATACGGCTGGTCAATACGCTACATCTGCTTGGAACTCTGCTTCCGCTGCGCATCAATCTGAgg                                                                                                                                                                                                                                              <  1:531688/301‑1 (MQ=255)`  
                                                               `ttGTGAACCTAGCGAACGCCGTGGATGACCGCGATGCTGTTCCGTTTGGTCAACTAAAGACCATGGACCAGAACTCATGGCAAGCACGTAATGAAGCCTTACAGTTCCGTAATGAGGCTGAGACTTTCAGAAACCAAGCGGAGGGCTTTAAGAACGAGTCCAGTACCAACGCTACGAACACAAAGCAGTGGCGCGATGAGACCAAGGGTTTCCGAGACGAAGCCGAGCGGTTCAAGAATACGGCTGGTCAATACGCTACATCTGCTGGGAACTCTGCTTCCGCTGCGCATCAATCTGAGGt                                                                                                                                                                                                                                             >  1:704201/1‑301 (MQ=255)`  
                                                                   `ctcggagaggtgtataagagacagccGCGATGCTGTTCCGTTTGGTCACCTAAAGACCATGGACCAGAACTCATGGCAAGCACGTAATGAAGCCTTACAGTTCCGTAATGAGGCTGAGACTTTCAGAAACCAAGCGGAGGGCTTTAAGAACGAGTCCAGTACAAACGCTACGAACACAAAGCAGTGGCGCGATGAGACCAAGGGTTTCCGAGACGAAGCCAAGCGGTTCAAGGATACGGCTGGTCAATACGCTACATCTGCTGGGAACTCTGCTTCCGCTGCGCATCAATCTGAGGTAAAc                                                                                                                                                                                                                                         <  1:807638/277‑1 (MQ=255)`  
                                                                         `aGCGAACGCCGTGGATGACCGCGATGCTGTTCCGTTTGGTCAACTAAAGACCATGGACCAGAACTCATGGCAAGCACGTAATGAAGCCTTACAGTTCCGTAATGAGGCTGAGACTTTCAGAAACCAAGCGGAGGGCTTTAAGAACGAGTCCAGTACCAACGCTACGAACACAAAGCAGTGGCGCGATGAGACCAAGGGTTTCCGAGACGAAGCCAAGCGGTTCAAGGATACGGCTGGTCAATACGCTACATCTGCTGGGAACTCTGCTTCCGCTGCGCATCAATCTGAGGTAAACGCTgag                                                                                                                                                                                                                                   >  1:332008/1‑301 (MQ=255)`  
                                                                               `cGCCGTGGATGACCGCGATGCTGTTCCGTTTGGTCAACTAAAGACCATGGACCAGAACTCATGGCAAGCACGTAATGAAGCCTTACAGTTCCGTAATGAGGCTGAGACTTTCAGAAACCAAGCGGAGGGCTTTAAGAACGAGTCCAGTACCAACGCTACGAACACAAAGCAGTGGCGCGATGAGACCAAGGGTTTCCGAGACGAAGCCAAGCGGTTCCAGGATACGGCTGCTCAATCCGCTACATCTGCTGGGAACTCTGCTTCCGCTGCGCATCCATCTGAGGTAAACGCTGAGAActct                                                                                                                                                                                                                             >  1:673032/1‑301 (MQ=255)`  
                                                                                 `cagTGGATGACCGCGATGCTGTTCCGTTTGGTCAACTAAAGACCATGGACCAGAACTCATGGCAAGCACGTAATGAAGCCTTACAGTTCCGTAATGAGGCTGAGACTTTCAGAAACCAAGCGGAGGGATTTAAGAACGAGTCCAGTACCAACGCTACGAACACAAAGCAGTGGCGCAATGAGACCAAGGGTTTCCGAGACGAAGCCAAGCGGTTCAAGAATACGGCTGGTCAATACGCTACATCTGCTGGGAACTCTGCTTCCGCTGCGAATCAATCTGAGGTAAACGCTGAGAACTATGc                                                                                                                                                                                                                           <  1:713046/299‑1 (MQ=255)`  
                                                                                     `ggATGACCGCGATGCTGTTCCGTTTGGTCAACTAAAGACCATGGACCAGAACTCATGGCAAGCACGTAATGAAGCCTTACAGTTCCATAATGAGGCTGAGACTTTCAGAAACCAAGCGGAGGGCTTTAAGAACGAGTCCAGTACCAACGCTACGAACACAAAGCAGTGGCGCGATGAGACCAAGGGTTTCCGAGACGAAGCCGAGCGGTTCAAGAATACGGCTGGTCAATACGCTACATCTGCTGGGAACTCTGCTTCCGCTGCGCATCAATCTGAGGTAAACGCTGAGAAcctgtctctt                                                                                                                                                                                                                       >  1:440086/1‑292 (MQ=255)`  
                                                                                        `tGACCGCGATGCTGTTCCGTTTGGTCAACTAAAGACCATGGACCAGAACTCATGGCAAGCACGTAATGAAGCCTTACGGTTCCGTAATGAGGCTGAGACTTTCAGAAACCAAGCGGAGGGCTTTAAGAACGAGTCCAGTACCAACGCTACGAACACAAAGCAGTGTCGCGATGAGACCAAGGGTTTCCGAGACGAAGCCAAGCGGTTCAAGAATACGGCTGGTCAATACGCTACATCTGCTGGGAACTCTGCTTCCGCTGCGCATCAATCTGAGGTAAACGCTGAGAACTCTGCCACAGCa                                                                                                                                                                                                                    <  1:273880/301‑1 (MQ=255)`  
                                                                                                 `ggctcggagatgtgtataagagacagccATGGACCAGAACTCATGGCAAGCACGTAATGAAGCCTTACAGTTCCGTAATGAGGCTGAGACTTTCAGAAACCAAGCGGAGGGCTTTAAGAACGAGTCCAGTACCAACGCTACGAACACAAAGCAGTGGCGCGATGAGACCAAGGGTTTCCGAGACGAAGCCAAGCGGTTCAAGGATACGGCTGGTCAATACGCTACATCTGCTGGGAACTCTGCTTCCGCTGCGCATCAATCTGAGGTAAACGCTGAGAACTCTGCCACAGCAGCCGCTAAc                                                                                                                                                                                                           <  1:16677/275‑1 (MQ=255)`  
                                                                                                       `tataagagacagaCTAAAGACCATGGACCAGAACTCATGGCAAGCACGTAATGAAGCCTTACAGTTCCGTAATGAGGCTGAGACTTTCAGAAACCAAGCGGAGGGCTTTAAGAACGAGTCCAGTACCAACGCTACGAACACAAAGCAGTGGCGCGATGAGACCAAGGGTTTCCGAGACGAAGCCAAGCGGTTCAAGGATACGGCTGGTCAATACGCTACATCTGCTGGGAACTCTGCTTCCGCTGCGCATCAATCTGAGGTAAACGCTGAGAACTATGCCACAGCATCCGCTAACTCTGCt                                                                                                                                                                                                     <  1:458433/289‑1 (MQ=255)`  
                                                                                                                   `aCTAAAGACCATGGACCAGAACTCATGGCAAGCACGTAATGAAGCCTTACAGTTCCGTAATGAGGCTGAGACTTTCAGAAACCAAGCGGAGGGCTTTAAGAACGAGTCCAGTACCAACGCTACGAACACAAGGCAGTGGCGCGATGAGACCAAGGGTTTCCGAGACGAAGCCGAGCGGTTCAAGAATACGGCTGGTCAATACGCTACATCTGCTGGGAACTCTGCTTCCGCTGCGCATCAATCTGAGGTAAACGCTGAGAACTATGCCACAGCATCCGCTAAcctgtctcttatacacatc                                                                                                                                                                                         >  1:296181/1‑283 (MQ=255)`  
                                                                                                                   `aCTAAAGACCATGGACCAGAACTCATGGCAAGCACGTAATGAAGCCTTACAGTTCCGTAATGAGGCTGAGACTTTCAGAAACCAAGCGGAGGGCTTTAAGAACGAGTCCAGTACCAACGCTACGAACACAAAGCAGTGGCGCGATGAGACCAAGGGTTTCCGAGACGAAGCCTAGCGGTTCAAGAATACGGCTGGTCAATACGCTACATCTGCTGGGAACTCTGCTTCCGCTGCGCATCAATCTGAGGTAAACGCTGAGAACTCTGCCACAGCATCCGCTAACTATGCCCATTTGGCAGaa                                                                                                                                                                                         >  1:240939/1‑301 (MQ=255)`  
                                                                                                                        `aGACCATGGACAAGAACTCATGGCAAGAACGTAATGAAGCCTTACAGTTCCGTAATGAGGCTGAGACTTTCAGAAACCAAGCGGAGGGCTTTAAGAACGAGTCCAGTACCAACGCTACGAACACAAAGCAGTGGAGCGATGAGACCAAGGGTTTCCGAGACGAAGCCGAGCGGTTCAAGAATACGGCTGGTCAATACGCTACATCTGCTGGGAACTCTGCTTCCGCTGCGCATCAATCTGAGGTAAACGCTGAGAACTCTGCCACAGCATCCGCTAACTCTGCTCATTTGGCAGAACagca                                                                                                                                                                                    <  1:427938/301‑1 (MQ=255)`  
                                                                                                                               `ggaCCAGAACTCATGGCAAGCACGTAATGAAGCCTTACAGTTCCGTAATGAGGCTGAGACTTTCAGAAACCAAGCGGAGGGCTTTAAGAACGAGTCCAGTACCAACGCTACGAACACAAAGCAGTGGCGCGATGAGACCAAGGGTTTCCGAGACGAAGCCAAGCGGTTCAAGAATACGGCTGGTCAATACGCTACATCTGCTGGGAACTCTGCTTCCGCTGCGCATCAATCTGAGGTAAACGCTGAGAACTCTGCCACAGCATCCGCTAAATCTGCTCATTTGGCAGAACAGCAAGAAGAc                                                                                                                                                                             >  1:652166/3‑301 (MQ=255)`  
                                                                                                                                             `ggCAAGCACGTAATGAAGCCTTATAGTTCCGTAATGAGGCTGAGACTTTCAGAAACCAAGCGGAGGGCTTTAAGAACGAGTCCAGTACCAACGCTACGAACACAAAGCAGTGGCGCGATGAGACC‑‑GGGTTTCCGAGACGAAGCCAAGTGGTTCAAGGATACGGCTGGTCAATACGCTACATCTGCTGGGAACTCTGCTTCCGCTGCTCATCAATCTGAGGTAAACGCTGAGAACTCTGCCATAGCAGCCGCTAACTCTGCTCATTTGGCAGAACAGCAAGCAGACCGTGCGGAACGTGAgg                                                                                                                                                             <  1:182557/301‑1 (MQ=255)`  
                                                                                                                                                 `aGCATGTAATGAAGCCTTACAGTTCCGTAATGAGGCTGAGACTTTCAGAAACCAAGCGGAGGGCTTTAAGAACGAGTCCAGTACCAACGCTACGAACACAAAGCAGTGGCGCGATGAGACCAAGGGTTTCCGAGACGAAGCCAAGCGGTTCAAGAATACGGCTGGTCAATACGCTACATCTGCTTGGACATCTGCTTCCGCTGCGCATCAATCTGAGGTAAACGCTGAGAACTATGCTACAGCATCCGCTAACTATGCTCATTTGGCAGAACAGCAAGCAGACCGTGCGGAACGTGAGGCa                                                                                                                                                           >  1:148790/1‑301 (MQ=255)`  
                                                                                                                                                       `tAATGAAGCCTTACAGTTCCGTAATGAGGCTGAGACTTTCAGAAACCAAGCGGAGGGCTTTAAGAACGAGTCCAGTACCAACGCTACGAACACAAAGCAGTGGCGCGATGAGACCAAGGGTTTCCGAGACGAAGCCAAGCGGTTCAAGAATACGGCTGGTCAATACGCTACATCTGCTTGGAACTCTGCTTCCGCTGCGCATCAATCTGAGGTAAACGCTGAGAACTATGCCACAGCATCCGCTAACTATGCTCATTTGGCAGAACAGCAAGCAGACAGTGCGGAACGTGAGGCAGACAAg                                                                                                                                                     >  1:694565/1‑301 (MQ=255)`  
                                                                                                                                                       `tAATGAAGCCTTACAGTTCCGTAATGAGGCTGAGACTTTCAGAAACCAAGCGGAGGGCTTTAAGAACGAGTCCAGTACAAACGCTACGAACACAAAGCAGTGGCGCGATGAGACCAAGGGTTTCCGAGACGAAGCCAAGCGGTTCAAGAATATGGCTGGTCAATACGCTACATCTGCTGGGAACTCTGCTTCCGCTGCGCATCAATCTGAGGTAAACGCTGAGAACTCTGCCACAGCATCAGCTAACTATGCTCATTTGGCAGAACAGCAAGCAgctgtatcttatacacatctccgagcc                                                                                                                                                     >  1:744262/1‑275 (MQ=255)`  
                                                                                                                                                       `tAATGAAGCCTTACAGTTCCGTAATGAGGCTGAGACTTTCAGAAACCAAGCGGAGGGCTTTAAGAACGAGTCAAGTACCAACGCTACGAACACAAAGCAGTGGCGCGATGAGACCAAGGGTTTCCGAGACGAAGCCAAGCGGTTCAAGGATACGGCTGGTCAATACGCTACATCTGCTGGGAACTCTGCTTCCGCTGCGCATCAATCTGAGGTAAACGCTGAGAACTCTGCCACAGCATCCGCTAACTATGCTCATTTGGCAGAACAGCAAGCAGACCGTGCGGAACGTGAGGCAGAcctt                                                                                                                                                     >  1:776813/1‑298 (MQ=255)`  
                                                                                                                                                        `aaTGAAGCCTTACAGTTCCGTAATGAGGCTGAGACTTTCAGAAACCAAGCGGAGGGCTTTAAGAACGAGTCCAGTACCAACGCTACGAACACAAAGCAGTGGAGCGATGAGACCAAGGGTTTCCGAGACGAAGCCAAGCGGTTCAAGAATACGGCTGGTCAATACGATACATCTGCTGGGAACTCTGCTTCCGCTGCGCATCAATCTGAGGTAAACGCTGAGAACTCTGCAACAGTAGCCGCTAACTATGCTCATTTGGCAGAACAGCAAGCAGACCGTGCGGAACGTGAGGCAGACAAGc                                                                                                                                                    >  1:228514/1‑301 (MQ=255)`  
                                                                                                                                                              `gCCTTACAGTTCCGTAATGAGGCTGAGACTTTCAGAAACCAAGCGGAGGGCTTTAAGAACGAGTCCAGTACCAACGCTACGAACACAAAGCAGTGGCGCGATGAGACCAAGGGTTTCCGAGACGAAGCCAAGCGGTTCAAGGATACGGCTGGTCAATACGCTACATCTGCTGGGAACTCTGCTTCCGCTGCGCATCAATCTGAGGTAAACGCTGAGAACTCTGCCACAGCATCCGCTAACTCTGCTCATTTGGCAGAACAGCAAGCAGACCGTGCGGAACGTGAGGCAGACAAGCTGGaaa                                                                                                                                              >  1:323561/1‑301 (MQ=255)`  
                                                                                                                                                               `ccTTACAGTTCCGTAATGAGGCTGAGACTTTCAGAAACCAAGCGGAGGGCTTTAAGAACGAGTCCAGTACCAACGCTACGAACACAAAGCAGTGGCGCGATGAGACCAAGGGTTTCCTAGACGAAGCCAAGCGGTTCAAGAATATGGCTGGTCAATACGCTACATCTGCTGGGAACTCTGCTTCCGCTGCGCATCAATCTGAGGTAAACGCTGAGAACTATGCCACAGCATCCGCTAACTCTGCTCATTTGGCAGAACAGCAAGCAGACCgctgtctcttatacacatctccgagcccacg                                                                                                                                             >  1:178522/1‑271 (MQ=255)`  
                                                                                                                                                                 `ttACAGTTCCGTAATGAGGCTGAGACTTTCAGAAACCAAGCGGAGGGCTTTAAGAACGAGTCCAGTACCAACGCTACGAACACAAAGCAGTGGCACGATGAGACCAAGGGTTTCCGAGACGAAGCCAAGCGGTTCAAGGATACGGCTGGTCAATACGCTACATCTGCTGGGAACTCTGCTTCCGCTGCGCATCAATCTGAGGTAAACGCTGAGAACTCTGCCACAGCATCCGCTAACTCTGCTCATTTGGCAGAACAGCAAGCAGACCGTGCGAAACGTGAGGCAGACAAGCTGGAAAAtt                                                                                                                                           >  1:549121/1‑301 (MQ=255)`  
                                                                                                                                                                      `gTTCCGTAATGAGGCTGAGACTTTCAGAAACCAAGCGGAGGGCTTTAAGAACGAGTCCAGTACCAACGCTACGAACACAAAGCAGTGGCGCGATGAGACCAAGGGTTTCCGAGACGAAGCCAAGCGGTTCAAGGATACGGCTGGTCAATACGCTACATCTGCTGGGAACTCTGCTTCCGCTGCGCATCAATCTGAGGTAAACGCTGAGAACTCTGCCACAGCATCCGCTAACTATGCTCATTTGGCAGAACAGCAAGCAGACCGTGCGGAACGTGAGGCAGACAAGCTGGAAAATTACAAt                                                                                                                                      >  1:658756/1‑301 (MQ=255)`  
                                                                                                                                                                      `gTTCCGTAATGAGGCTGAGACTTTCAGAAACCAAGCGGAGGGCTTTAAGAACGAGTCCAGTACCAACGCTACGAACACAAAGCAGTGGCGCGATGAGACCAAGGGTTTCCGAGACGAAGCCAAGCGGTTCAAGGATACGGCTGGTCAATACGCTACATCTGCTGGGAACTCTGCTTCCGCTGCGCATCAATCTGAGGTAAACGCTGAGAACTATGCCACAGCATCCGCTAACTCTGCTCATTTGGCAGAACAGCAAGCAGACCGTGCGGAACCTgtctcttatacacatctccgagcccac                                                                                                                                      >  1:123395/1‑275 (MQ=255)`  
                                                                                                                                                                        `tCCGTAATGAGGCTGAGACTTTCAGAAACCAAGTGGAGGGCTTTAAGAACGAGTCCAGTACCAACGCTACGAACACAAAGCAGTGGCTCGATGAGACCAAGGGTTTCCGAGACGAAGCCGAGCGGTTCAAGAATACGGCTGGTCAATACGCTACATCTGCTGGGAACTCTGCTTCCGCTGCGCATCAATCTGAGGTAAACGCTGAGAACTCTGCCACAGCATCCGCTAACTATGCTCATTTGGCAGAACAGCAAGCAGACCGTGCGGAACGTGAGGCAGACAAGCTGGAAAATTACAATgg                                                                                                                                    >  1:575247/1‑301 (MQ=255)`  
                                                                                                                                                                        `tCCGTAATGAGGCTGAGACTTTCAGAAACCAAGCGGAGGGCTTTAAGAACGAGTCCAGTACCAACGCTACGAACACAAAGCAGTTGCTCGATGAGACCAAGGGTTTCCGAGACGAAGCCAAGCGGTTCAAGGATACGGCTGGTCAATACGCTACATCTGCTGGGAACTCTGCTTCCGCTGCGCATCAATCTGAGGTAAACGCTGAGAACTCTGCCACAGCATCCGCTAACTCTGCTCATTTGGCATAACAGCAAGCAGACCGTGCGAAACGTGAGGCAGACAAGCTTGAAAATTACAATgg                                                                                                                                    >  1:799899/1‑301 (MQ=255)`  
                                                                                                                                                                               `agAGGGTGAGACTTTCAGAAACCAAGCGGAGTGCTCTACGAACGAGTCCAGTACCAACGCCACGAACACAAAGCAGTGGCGCGATGAGACCAAGGGTTTCCGAGACGAAGCCGAGCGGTTCAAGAATACGGCTGGTCAATACGCTACATCTGCTGGGAACTCTGCTTCCGCTGCGCATCAATCTGAGGTAAACGCTGAGAACTCTGCCACAGCAGCCGCTAACTCTGCTCATTTGGCAGAACAGCAAGCAGACCGTGCGGAACGTGAGGCAGACAAGCTGGAAAATTACAATGGATTGGCt                                                                                                                             <  1:21908/300‑1 (MQ=255)`  
                                                                                                                                                                                  `ggCTGAGACTTTCAGAAACCAAGCGGAGGGCTTTAAGAACGAGTCCAGTACCAACGCTACGAACACAAAGCAGTGGCGCGATGAGACCAAGGGTTTCCGAGACGAAGCCAAGCGGTTCAAGAATACGGCTGGTCAATACGCTACATCTGCTTGGAACTCTGCTTCCGCTGCACATCAATCTGAGGTAAACGCTGAGAACTATGCCACAGCATCCGCTAACTATGCTCATTTGGCAGAACAGCAAGCAGACCGTGCGGAACGTGAGGCAGACAAGCTGGAAAATTACAATGGATTGGCTGGt                                                                                                                          >  1:483770/1‑301 (MQ=255)`  
                                                                                                                                                                                  `ggCTGAGACTTTCAGAAACCAAGCGGAGGGCTTTAAGAACGAGTCCAGTACCAACGCTACGAACACAAAGCAGTGGCGCGATGAGACCAAGGGTTTCCAAGACGAAGCCAAGCGGTTCAAGAATACGGCTGGTCAATACGCTACATCTGCTTGGAACTCTGCTTCCGCTGCGCATCAATCTGAGGTAAACGCTGAGAACTATGCCACAGAATCCGCTAACTATGCTCATTTGGCAGAACAGCAAGCAGACCGTGCGGAACGTGAGGCAGACAAGCTGGAAAATTACAATGGATTGGCTggg                                                                                                                          >  1:81623/1‑300 (MQ=255)`  
                                                                                                                                                                                          `cTTTCAGAAACCAAGCGGAGGGCTTTAAGAACGAGTCCAGTACCAACGCTACGAACACAAAGCAGTGGCGCGATGAGACCAAGGGTTTCCGAGACGAAGCCGAGCGGTTCAAGAATACGGCTGGTCAATACGCTACATCTGCTGGGAACTCTGCTTCCGCTGCGCATCAATCTGAGGTAAACGCTGAGAACTCTGCCACAGCATCCGCTAACTATGCTCATTTGGCAGAACAGCAAGCAGACCGTGCGGAACGTGAGGCAGACAAGCTGGAAAATTACAATGGATTGGCTGGTGCAATTGa                                                                                                                  >  1:71949/1‑301 (MQ=255)`  
                                                                                                                                                                                            `ttCAGAAACCAAGCGGAGGGCTTTAAGAACGAGTCCAGTACCAACGCTACGAACACAAAGCAGTGGCGCGATGAGACCAAGGGTTTCCGAGACGAAGCCGAGCGGTTCAAGAATACGGCTGGTCAATACGCTACATCTGCTGGGAACTCTGCTTCCGCTGCGCATCAATCTGAGGTAAACGCTGAGAACTCTGCCACAGCATCCGCTAACTATGCTCATTTGGCAGAACAGCAAGCAGACCGTGCGGAACGTGAGGCAGACAAGCTGGAAAATTACAATGGATTGGCTGGTGCAATTGATa                                                                                                                >  1:664817/1‑301 (MQ=255)`  
                                                                                                                                                                                               `aGAAACCAAGCGGAGTGCTTTAAGAACGAGTCCAGTACCAACGCTACGAACACAAAGCAGTGGCGCGATGAGACCAAGGGTTTCCGAGACTAAGCCAAGCGGTTCAAGAATACGGCTGGTCAATACGCTACATCTGCTTTAAACTCTGCTTCCGCTGTGCATCAATCTGAGGTAAACGCTGAGAACTATGCCACAGCATCCGCTAACTATGCTCATTTGGCAGAACAGCAAGCAGACCGTGCTGAACGTGAGGCAGACAAGCTGGAAAATTACAATGGATTGGCTGGTGCAATTGATAAgg                                                                                                             <  1:28371/301‑1 (MQ=255)`  
                                                                                                                                                                                                `aaaaCCAAGCGGAGGGCTTTAAGAACGAGTCCAGTACCAACGCTACGAACACAAAGCAGTGGCGCGATGAGACCAAGGGTTTCCGAGACGAAGCCAAGCGGTTCAAGGATACGGCTGGTCAATACGCTACATCTGCTGGGAACTCTGCTTCCGCTGCTCATCAATCTGAGGTAAACGCTGAGAACTCTGCCACAGCATCCGCTAACTATGCTCATTTGGCAGAACAGCAAGCAGACCGTGCGGAACGTGAGGCAGACAAGCTGGAAAATTACAATGGATTGGCTGGTGCAATTGATAAGGt                                                                                                            <  1:356660/300‑1 (MQ=255)`  
                                                                                                                                                                                                      `aaGCGGAGGGCTTTAAGAACGAGTCCAGTACCAACGCTACGAACACAAAGCAGTGGCGCGATGAGACCAAGGGTTTCCGAGACGAAGCCAAGCGGTTCAAGGATACGGCTGGTCAATACGCTACATCTGCTGGGAACTCTGCTTCCGCTGCGCATCAATCTGAGGTAAACGCTGAGAACTCTGCCACAGCAGCCGCTAACTCTGCTCATTTGGCAGAACAGCAAGCAGACCGTGCGGAACGTGAGGCAGACAAGCTGGAAAATTACAATGGATTGGCTGGTGCAATTGATAAGGTAGATgg                                                                                                      <  1:22589/301‑1 (MQ=255)`  
                                                                                                                                                                                                       `aGCGGAGGGCTTTAAGAACGAGTCCAGTACCAACGCTACGAACACAAAGCAGTGGCGCGATGAGACCAAGGGTTTCCGAGACGAAGCCGAGCGTTTCAAGAATACGGCTGGTCAATACGCTACATCTGCTGGGAACTCTGCTTCCGCTGCGCATCAATCTGAGGTAAACGCTGAGAACTCTTCCACAGCAGCCGCTAACTCTGCTCATTTGGCAGAACAGCAAGCAGACCGTGCGGAACGTGAGGCCGACAAGCTGGAAAATTACAATGGATTTGCTGGTGCAATTGATAAGGTAGATGGa                                                                                                     >  1:109759/1‑301 (MQ=255)`  
                                                                                                                                                                                                        `gCGGATGGCTTTAAGAACGAGTCCAGTACCAACGCTACGAACACAAAGCAGTGGCGCGATGAGACCAAGGGTTTCCGAGACGAAGCCGAGCGGTTCAAGAATACGGCTGGTCAATACGCTACATCTGCTGGGAACTCTGCTTCCGCTGCGCATCAATCTGAGGTAAACGCTGAGAACTATGCCACAGCATCCGCTAACTCTGCTCATTTGGCAGAACAGCAAGCAGACCGTGCGGAACGTGAGGCAGACAAGCTGGAAAATTACAATGGATTGGCTGGTGcctgtctcttatacacatctc                                                                                                    >  1:730845/1‑281 (MQ=255)`  
                                                                                                                                                                                                          `ggAGGGCTTTAAGAACGAGTCCAGTACCAACGCTACGAACACAAAGCAGTGGCGCGATGAGACCAAGGGTTTCCGAGACGAAGCCAAGCAGTTCAAGAATACGGCTGGTCAATACGCTACATCTGCTTGGAACTCTGCTTCCGCTGCGCATCAATCTGAGGTAAACGCTGAGAACTAGGCCACAGCATCCGCTAACTATGCTCATTTGGCAGAACAGCAAGCAGACCGTGCGGAACGTGAGGCAGACAAGCTGGAAAATTACAATGGATTGGCTGGTGCAATTGATAAGGTAGATGGAAcc                                                                                                  <  1:401695/301‑1 (MQ=255)`  
                                                                                                                                                                                                             `gggCTTTAAGAACGAGTCCAGTACCAACGCTACGAACACAAAGCAGTGGCGCGATGAGACCAAGGGTTTCCGAGACGAAGCCAAGCGGTTCAAGGATACGGCTGGTCAATACGCTACATCTGCTGGGAACTCTGCTTCCGCTGCGCATCAATCTGAGGTAAACGCTGAGAACTCTGCCACAGCAGCCGCTAACTCTGCTCGTTTGGCAGAACAGCAAGCAGACCGTGCGGAACGTGAGGCAGACAAGCTGGAAAATTACAATGGATTGGCTGGTGCAATTGATAAGGTAGATGGAACCAAt                                                                                               >  1:623688/1‑301 (MQ=255)`  
                                                                                                                                                                                                              `ggCTTTAAGAACGAGTCCAGTACCAACGCTACGAACACAAAGCAGTGGTGCGATGAGACCAAGGGTTTCCGAGACGAAGCCAAGCGGTTCAAGGATACGGCTGGTCAATACGCTACATCTGCTGGGAACTCTGCTTCCGCTGCGCATCAATCTGAGGTAAACGCTGAGAACTCTGCCACAGCATCCGCTAACTATGCTCATTTGGCAGAACAGCAAGCAGACCGTGCGGAACGTGAGGCAGACAAGCTGGAAAATTACAATGGATTGGCTGGTGCAATTGATAAGGTAGATGGAACCAAtg                                                                                              <  1:734056/301‑1 (MQ=255)`  
                                                                                                                                                                                                               `gCTTTAAGAACGAGTCCAGTACCAACGCTACGAACACAAAGCAGTGGCGCGATGAGACCAAGGGTTTCCGAGACGAAGCCAAGCGGTTCAAGGATACGGCTGGTCAATACGCTACATCTACTGGGAACTCTGCTTCCGCTGCGCATCAATCTGAGGTAAACGCTGAGAACTCTGCCACAGCATCCGCTAACTATGCTCATTTGGCAGAACAGCAAGCAGACCGTGCGGAACGTGAGGCAGACAAGCTGGAAAATTACAATGGATTGGCTGGTGCAATTGATAAGGTAGATGGAACCAAtgt                                                                                             >  1:50541/1‑301 (MQ=255)`  
                                                                                                                                                                                                                    `gtataagagacaggTACCAACGCTACGAACACAAAGCAGTGGCGCGATGAGACCAAGGGTTTCCGAGACGAAGCCGAGCGGTTCAAGAATACGGCTGGTCAATACGCTACATCTGCTGGGAACTCTGCTTCCGCTGCGCATCAATCTGAGGTAAACGCTGAGAACTCTGCCATAGCATCCGCTAACTCTGCTCATTTGGCAGAACAGCAAGCAGACCGTGCGGAACGTGAGGCAGACAAGCTTGAAAATTACAATGGATTGGCTGGTGCAATTGATAAGGTAGATGGAACCAATGTGTACt                                                                                        <  1:435492/288‑1 (MQ=255)`  
                                                                                                                                                                                                                      `gAACGAGTCCAGTACCAACGCTACGAACACAAAGCAGTGGCGCGATGAGACCAAGGGTTTCCGAGATGAAGCCAAGCGGTTCAAGGATACGGCTGGTCAATACGCTACATCTGCTGGGAACTCTGCTTCCGCTGCGCATCAATCTGAGGTAAACGCTGAGAACTATGCCACAGCATCCGCTAACTCTGCTCATTTGGCAGAACAGCAAGCAGACCGTGCGGAACGTGAGGCAGACAAGCTGGAAAATTACAATGGATTGGCTGGTGCAATTGATAAGGTAGATGGAACCAATGTGTACTgg                                                                                      <  1:390888/301‑1 (MQ=255)`  
                                                                                                                                                                                                                        `gtataagagacagcAACGCTACGAACACAAAGCAGTGGCGCGATGAGACCAAGGGTTTCCTAGACGAAGCCGAGCGGTTCAAGAATACGGCTGGTCAATACGCTACATCTGCTGGGAACTCTGCTTCCGCTGAGCATCAATCTGAGGTAAACGCTGAGAACTCTGCCACAGCATCCGCTAACTATGCTCATTTGGCAGAACAGCAAGCAGACCGTGCGGAACGTGAGGCAGACAAGCTGGAAAATTACAATGGATTGGCTGGTGCAATTGATAAGGTAGATGGAACCAATGTGTACTggaa                                                                                    <  1:580518/288‑1 (MQ=255)`  
                                                                                                                                                                                                                            `gTCCAGTACCAACGCTACGAACACAAAGCAGTGGCGCGATGAGACCAAGGGTTTCCGAGACGAAGCCAAGTGGTTCAAGGATACGGCTGGTCAATACGCTACATCTGCTGGGAACTCTGCTTCCGCTGCGCATCAATCTGAGGTAAACGCTGAGAACTCTGCCACAGCATCCGCTAACTATGCTCATTTGGCAGAACAGCAAGCAGACCGTGCGGAACGTGAGGCAGACCAGCTGGAAAATTACAATGGATTGGCTGGTGCAATTGATAAGGTAGATGGAACCAATGTGTACTggaaagga                                                                                >  1:190191/1‑301 (MQ=255)`  
                                                                                                                                                                                                                            `gTCCAGTACCAACGCTACGAACACAAAGCAGTGGCGCGATGAGACCAAGGGTTTCCGAGACGAAGCCAAGCGGTTCAAGAATACGGCTGGTCAATACGCTACATCTGCTTGGAACTCTGCTTCCGCTGCGCATCAATCTGAGGTAAACGCTGAGAACTATGCCACAGCATCCGCTAACTATGCTCATTTGGCAGAACAGCAAGCAGACCGTGCGGAACGTGAAGCAGACAAGCTGGAAAATTACAATGGATTGGCTGGTGCAATTGATAAGGTAGATGGAACCAATGTGTACTggaaagga                                                                                >  1:377968/1‑301 (MQ=255)`  
                                                                                                                                                                                                                             `tCCAGTACCAACGCTACGAACACAAAGCAGTGGCGCGATGAGACCAAGGGTTTCCGAGACGAAGCCGAGCGGTTCAAGAATACGGCTGGTCAATACGCTACATCTGCTGGGAACTCTGCTTCCGCTGCGCATCAATCTGAGGTAAACGCTGAGAACTCTGCCACAGCATCCGCTAACTATGCTCATTTGGCAGAACAGCAAGCAGACCGTGCGGAACGTGAGGCAGACAAGCTGGAAAATTACAATGGATTGGCTGGTGCAATTGATAAGGTAGATGGAACCAATGTGTACTggaaaggaa                                                                               >  1:535245/1‑301 (MQ=255)`  
                                                                                                                                                                                                                             `tCCAGTACCAACGCTACGAACACAAAGCAGTGGCGCGATGAGACCAAGGGTTTCCGAGACGAAGCCGAGCGGTTCAAGAATACGGCTGGTCAATACGCTACATCTGCTGGGAACTCTGCTTCCGCTGCGCATCAATCTGAGGTAAACGCTGAGAACTCTGCCACAGCATCCGCTAACTATGCTCATTTGGCAGAACAGCAAGCAGACCGTGCGGAACGTGAGGCAGACAAGCTGGAAAATTACAATGGATTGGCTGGTGCAATTGATAAGGTAGATAGAAccctgtctcttatacacatct                                                                               >  1:700768/1‑282 (MQ=255)`  
                                                                                                                                                                                                                                 `gTACCAACGCTACGAACACAAAGCAGTGGCGCGATGAGACCAAGGGTTTCCGAGACGAAGCCAAGCGGTTCAAGGATACGGCTGGTCAATACGCTACATCTGCTGGGAACTCTGCTTCCGCTGCGCATCAATCTGAGGTAAACGCTGAGAACTCTGCCACAGCATCCGCTAACTTTGCTCATTTGGCAGAACAGCAAGCAGACCGTGCGGAACGTGAGGCAGACAAGCTGGAAAATTACAATGGATTGGCTGGTGCAATTGATAAGGTAGCTgtctcttatacacatctccgagcccacga                                                                           >  1:676902/1‑273 (MQ=255)`  
                                                                                                                                                                                                                                           `tACGAACACAAAGCAGTGGCGCGATGAGACCCAGGGTTTCCGAGACGAAGCCAAGCGGTTCAAGAATACGGCTGGTCAATACGCTACATCTGCTGGGAACTCTGCTTCCGCTTCGCATCAATCTGAAGTAAACGCTGAGAACTCTGCCACAGCATCCGCTAACTATGCTCATTTGGCAGAACAGCAAGCAGACCGTGCGGAACGTGAGGCAGACAAGCTGGAAAATTACAATGGATTGGCTGGTACAATTGATAAGGTAGATGGAACCAATGTGTACTGGAAAGGAAATATTCACGCTAAc                                                                 >  1:643637/1‑301 (MQ=255)`  
                                                                                                                                                                                                                                              `tgtataagagacagggCGCGATGAGACCAAGGGTTTCCGAGACGAAGCCAAGCGGTTCAAGGATACGGCTGGTCAATACGCTACATCTGCTGGGAACTCTGCTTCCGCTGCGCATCAATCTGAGGTAAACGCTGAGAACTCTGCCACAGCATCCGCTAACTTTGCTCATTTGGCAGAACAGCAAGCAGACCGTGCGGAACGTGAGGCAGACAATCTGGAAAATTACAATGGATTGGCTGGTGCAATTGATAAGGTAGATGGAACCAATGTGTACTGGAAAGGAAATATTCACGCTAACggg                                                              <  1:210189/287‑1 (MQ=255)`  
                                                                                                                                                                                                                                              `gAACACAAAGCAGTGGCGCGATGAGACCAAGGGTTTCCGAGACGAAGCCGAGCGGTTCAAGAATACGGCTGGTCAATACGCTACATCTGCTGGGAACTCTGCTTCCGCTGCGCATCAATCTGAGGTAAACGCTGAGAACTCTGCCACAGCATCCGCTAACTATGCTCATTTGGCAGAACAGCAAGCAGACCGTGCGGAACGTGAGGCAGACAAGCTGGAAAATTACAATGGATTGGCTGGTGCAATTGATAAGGTAGATGGAACCAATGTGTACTGGAAAGGAAATATTCACGCTAACggg                                                              >  1:388684/1‑301 (MQ=255)`  
                                                                                                                                                                                                                                                `acacAAAGCAGTGTCGCGATGAGACCAAGGGTTTCCGAGACGAAGCCGAGCGGTTCAAGAATACGGCTGGTCAATACGCTACATCTGCTGGGAACTCTGCTTCCGCTGCGCATCAATCTGAGGTAAAAGCTGATAACTATGCCACGGCATCCGCTAACTCTGCTCATTTGGCAGAACAGCAAGCAGACCGTGCGGAATGTGAGGCAGACAAGCTGGAAAATTACAATGGATTGGCTGGTGCAATTGATAAGGTAGATGGAACCAATGTGTACTGGAAAGGAAATATTCACGCTAACGGgcg                                                            >  1:315074/1‑301 (MQ=255)`  
                                                                                                                                                                                                                                                `acacAAAGCAGTGGCGCGATGAGACCAAGGGTTTCCGAGACGAAGCCGAGCGGTTCAAGAATACGGCTGGTCAATACGCTACATCTGCTGGGAACTCTGCTTCCGCTGCGCATCAATCTGAGGTAAACGCTGAGAACTCTGCCACAGCATCCGCTAACTCTGCTCATTTGGCAGAACAGCAAGCAGACCGTGCGAAACGTGAGGCAGACAAGCTGGAAAATTACAATGGATTGGCTGGTGCAATTGATAAGGTAGATGGAACCAATGTGTACTGGAAAGGAAATATTCACGCTAACGTgcg                                                            >  1:555320/1‑301 (MQ=255)`  
                                                                                                                                                                                                                                                 `cacaAAGCAGTGTCGCGATGAGACAAAGGGTTTCCGAGACGAAGCCGAGCGGTTCAAGAATACGGCTGGTCAATACGCTACATCTGCTGGGAACTCTGCTTCCGCTGCGCATCAATCTGAGGTAAACGCTGAGAACTCTGCCACAGCATCCGATAACTATGCTAATTTGGCAGAACAGCAAGCAGACCGTGCGGAACGTGAGGCAGACAAGCTGGAAAATTACAATGGATTGGCTGGTGCAATTGATAAGGTAGATGGAACCAATGTGTACTGGAAAGGAAATATTCACGCTAACGGgcgc                                                           >  1:26898/1‑301 (MQ=255)`  
                                                                                                                                                                                                                                                        `gagatgtgtataagagacaggggTTTCCGAGACGAAGCCAAGCGGTTCAAGAATACGGCTGGTCAATACGCTACATCTGCTAGGAACTCTGCTTCCGCTGCGCATCAATCTGAGGTAAACGCTGAGAACTCTGCCACAGCATCCGCTAACTATGCTCATTTGGCAGAACAGCAAGCAGACCGTGCGGAACATGAGGCAGACAAGCTGGAAAATTACAATGGATTGGCTGGTGCAATTGATAAGGTAGATGGAACCAATGTGTACTGGAAAGGAAATATTCACGCTAACGGGCGCCTTTACa                                                    <  1:311361/281‑1 (MQ=255)`  
                                                                                                                                                                                                                                                        `cAGTGGCGCGATGAGACCAAGGGTTTCCGAGACGAAGCCAAGCGGTTCAAGGATACGGCTGGTCAATACGCTACATCTGCTGGGAACTCTGCTTCCGCTGCGCATCAATCTGAGGTAAACGCTGAGAACTATGCCACAGCATCCGCTAACTCTGCTCATTTGGCAGAACAGCAAGCAGACGGTGCGGAACGTGAGGCAGACAAGCTGGAAAATTACAATGGATTGGCTGGTGCAATTGATAAGGTAGATGGAACCAATGTGTACTGGAAAGGAAATATTCACGCTAACGGGCGACTTTACa                                                    <  1:769942/301‑1 (MQ=255)`  
                                                                                                                                                                                                                                                        `agatgtgtataagagaCAGAGGGTTTCCGAGACGAAGCCAAGCGGTTCAAGGATACGGCTGGTCAATACGCTACATCTGCTGGGAACTCTGCTTCCGCTGCGCATCAATCTGAGGTAAACGCTGAGAACTCTGCCACAGCATCCGCTAACTATGCTCATTTGGCAGAACAGCAAGCAGACCGTGCGGAACGTGAGGCAGACAAGCTGGAAAATTACAATGGATTGGCTGGTGCAATTGATAAGGTAGATGGAACCAATGTGTACTGGAAAGGAAATATTCACGCTAACGGGCGCCTTTACa                                                    <  1:724663/289‑1 (MQ=255)`  
                                                                                                                                                                                                                                                         `aGTGGCGCGATGAAACCAAGGGTTTCCGAGACGAAGCCTAGGGGTTCAAGGATACGGCTGGTCAATACGCTACATCTGCTGGGAACTCTGCGTCCGCTGCGCATCAATCTGAGGTAAACGCTGAGAACTCTGCCACAGCATCCGCTAACTATGCTCATTTGGCAGAACAGCAAGCAGACCGTGCGGAACGTGAGGCAGACAAGCTGGAAAATTACAATTGATTGGCTGGTGCAATTGATAAGGTAGATGGAACCAATGTGTACTGGAAAGGAAATATTCACGCTAACGGGCGCCTTTACAt                                                   <  1:127560/301‑1 (MQ=255)`  
                                                                                                                                                                                                                                                          `gtgtCGCGATTAGACCAAGGGTTTCCGAGACGAAGCCAAGCGTTTCAAGAATACGGCTGGTCAATACGCTACATCTTCTTGGAACTCTGCTTCCGCTGCGCATCAATCTGAGGTAAACGCTGAGAACTATGCCACAGCATCCGCTAACTATGCTCATTTGGCAGAACAGCAAGCAGACCGTGCGGAACGTTAGGCAGACAAGCTGGAAAATTACAATGGATTGGCTGGTGCAATTGATAAGGTAGATGGAACCAATGTGTACTGGAAAGGAAATATTCACGCTAACGGGCGCCTTTACATg                                                  >  1:363575/1‑301 (MQ=255)`  
                                                                                                                                                                                                                                                            `ggCGTGATGAGACCAATGGTTTCCGAGACGAAGCCAAGCGGTTCAAGGATACGGCTGGTCAATACGCTACATCTGCTGTGAACTCTGCTTCCGCTGCGCATCAATCTGAGGTAAACGCTGAGAACTCTGCCACAGCATCCGCTAACTATGCTCATTTGGCAGAACAGCAAGCAGACCGTGCGGAACGTGAGGCAGACAAGCTGGAAAATTACAATGGATTGGCTGGTGCAATTGATAAGGTAGATGGAACCAATGTGTACTGGAAAGGAAatctgtctcttatacacatctccgagcccac                                                >  1:570980/1‑272 (MQ=255)`  
                                                                                                                                                                                                                                                            `ggCACGATGGGACCAAGGGGTTCCGAGAAGAAGCCAAGCGGTTCAAGAATACGGCTGGTCAATACGCTACATCTGGTTGGAACTCTGCTTCCGCTGCGCATCAATCTGAGGTAAACGCTGAGAACTATGCAACAGCATCCGCAAACTATGCTCATTGGGCAGAACAGCAAGCAGAACGTGAGGAACGTGAGGCAGACAAGATGGAAAATTACAATGGAATGGCTGGTGCAATTGATAAGGTAGATGGAACCAATGTGTACTGGAAAGGAAATATTCACGCTAACGGGCGCCTTTACAGGAc                                                <  1:467481/301‑1 (MQ=255)`  
                                                                                                                                                                                                                                                                   `ggatacaaaGGGTTGCCGATACGAAGCCAAGCGGTTTAAGGATTCGGCTGGTCAATACGCTCCATCTTCTGGTAACACTGCTTCCGCTTCCCATCAATCTGATGTAAACGCTGAAAACTATGCCACAGCATCCGCTAACTCTGCTCATTTGGCAGAACAGCAAGCAGACCGTGCGGAACTTGAGGCAGACAAGCTGGAAAATTACAATGGATTGGCTGGTGCAATTGATAAGGTAGATGGAACCAATGTGTACTGGAAAGGAAATATTCACGCTAACGGGCGCCTTTACATGACCACAAAc                                         <  1:152835/294‑1 (MQ=255)`  
                                                                                                                                                                                                                                                                   `agagaCCAAGGGTTTCCGAGACGAAGCCGAGCGGTTCAAGAATACGGCTGGTCAATACGCTACATCTTCTGGGAACTCTGCTTCCGCTGCGCATCAATCTGATGTAAACGCTGAGAACTCTGCCACAGCATCCGCTAACTATGCTCATTTGGCAGAACAGCAAGCAGACCGTGCGGAACGTGAGGCAGACAAGCTGGAAAATTACAATGGATTGGCTGGTGCAATTGATAATGTAGATGGAACCAATGTGTACTGGAAAGGAAATATTCACGCTAACGGGCGCCTTTACATGACCACAAAc                                         <  1:611559/300‑1 (MQ=255)`  
                                                                                                                                                                                                                                                                   `aagagacaggggTTTCCGAGACGAAGCCGAGCGGTTCAAGAATACGGCTGGTCAATACGCTACATCTGCTGGGAACTCTGCTTCCGCTGCGCATCAATCTGAGGTAAACGCTGAGAACTCTGCCACAGCATCCGCTAACTTTGCTCATTTGGCAGAACAGCAAGCAGACCGTGCGGAACGTGAGGCAGACAAGCTGGAAAATTACAATGGATTGGCTGGTGCAATTGATAAGGTAGATGGAACCAATGTGTACTGGAAAGGAAATATTCACGCTAACGGGCGCCTTTACATGACAACAAAc                                         <  1:245769/292‑1 (MQ=255)`  
                                                                                                                                                                                                                                                                      `gaCCAAGGGTTTCCGAGACGAAGCCGAGCGGGTCAAGAATACGGCTGGTCAATACGCTACATCTGCTGGGAACTCTGCTTCCGCTGCGCATCAATCTGAGGTAAACGCTGAGAACTATGCCACAGCATCCGCTAACTCTGCTCATTTGGCAGAACAGCAAGCAGACCGTGCGGAACGTTAGGCAGACAAGGTGGAAAATTACAATGGATTGGCTGGTGCAATTGATAAGGTAGAAGGAACCAATGTGTACTGGTAAGGAAATATTCACCCTAACGGGCGCCTTTCCATTGACACCAACggg                                      >  1:715915/1‑300 (MQ=255)`  
                                                                                                                                                                                                                                                                      `gaCCAAGGGTTTCCGAGACGAAGCCAAGCGGTTCAAGAATACGGCTTGTCAATACGCTACATCTGCTTGGAACTCTGCTTCCGCTGCGCATCAATCTGAGGTAAACGCTGAGAACTATGCCACAGCATCCGCTAACTATGCTCATTTGGCAGAACAGCAAGCAGACCGTGCGGAACGTGAGGCAGACAAGCTGGAAAATTACAATGGATTGGCTGGTGCAATTGATAAGGTAGATGGAACCAATGTGTACTGGAAAGGAAATATTCACGCTAACAGGCGCCTTTACATGACCACAAACGGt                                      >  1:448226/1‑301 (MQ=255)`  
                                                                                                                                                                                                                                                                      `gaCCAAGGGTTTCCGAGACGAAGCCAAGCGGTTCAAGAATACGGCTGGTCAATACGCTACATCTGCTTGGAACTCTGCTTCCGCTGCGCATCAATCTGAGGTAAACGCTGAGAACTATGCCACAGCATCCGCTAACTATGCTCATTTGGCAGAACAGCAAGCAGACCGTGCGGAACGTGAGGCCGACAAGCTGGAAACTTACAATGGATTGGCTGGTGCAATTGATAAGGTAGATGGACCCAATGTGTACTGGAAAGTAAATATTCACGCCACCGGGCGCCCTTCCATGACCACAAACGGt                                      >  1:812728/1‑301 (MQ=255)`  
                                                                                                                                                                                                                                                                            `gggTTTCCGAGACGAAGCCAAGCGGTTCAAGGATACGGCTTGTCAATACGCTACATCTGCTGGGAACTCTGCTTCCGCTGTGCATCAATCTGAGGTAAACGCTGAGAACTCTGCCACAGCAGCCGCTAACTCTTCTCATTTGGCAGAACAGCAAGCAGACCGTGCGGAACGTGAGGCAGACAAGCTGGAAAATTACAATGGATTGGCTGGTGCAATTGATAAGGTAGATGGAACCAATGTGTACTGGAAAGGAAATATTCACGCTAACGGGCGCCTTTACATGACcacctgtctcttataa                                >  1:279099/1‑288 (MQ=255)`  
                                                                                                                                                                                                                                                                            `gggTTTCCGAGACGAAGCCAAGCGGTTCAAGAATACGGCTGGTCAATACGCTACATCTGCTTGGAACTCTGCTTCCGCTGCGCATCAATCTGAGGTAAACGCTGAGAACTATGCCACAGCATCCGCTAACTATGCTCATTTGGCAGAACAGCAAGCAGACCGTGCGGAACGTGAGGCAGACAAGCTGGAAAATTACAATGGATTGGCTGGTGCAATTGATAAGGTAGATGGAACCAATGTGTACTGGAAAGGAAATATTCACGCTAACGGGCGccctgtctcttatacacatctccgagcc                                >  1:629699/1‑275 (MQ=255)`  
                                                                                                                                                                                                                                                                            `gggTTTCAGAGACGAAGCCAAGCGGTTCAAGAATACGGCTGGTCAATACGCTACATCTGCTGGGAACTCTGCTTCCGCTGCGCATCAATCTGAGGTAAACGCTGAGAACTATGCCACAGCATCCGCTAACTCTGCTAATTTGGCAGAACAGCAAGCAGACCGTGCGGAACGTGAGGCAGACAAGCTGGAAAATTACAATGGATTGGCTGGTGCAATTGATAAGGTAGATGGAACCAATGTGTACTGGAAAGGAAATATTCACGCTAACGGGCGCCTTTACATGACCACAAACGGTTTTGAc                                <  1:706031/301‑1 (MQ=255)`  
                                                                                                                                                                                                                                                                                     `agaCGAAGCCGAGCGGTTCAAGAATACGGCTGGTCAATACGCTACATCTGCTGGGAACTCTGCTTCCGCTGCGCATCAATCTGAGGTAAACGCTGAGAACTCTGCCACAGCATCCGCTAACTATTCTCATTTGGCAGAACAGCAAGCAGACCGTGCGGAACGTGAGGCAGACAAGCTGGAAAATTACAATGGATTGGCTGGTGCAATTGATAAGGTAGATGGAACCAATGTGTACTGGAAAGGAAATATTCAAGCTAACGGGCGCCTTTACATgctgtctcttatacacatctccgagccc                       >  1:290099/1‑274 (MQ=255)`  
                                                                                                                                                                                                                                                                                            `gCCGAGCGGTTCAAGAATACGGCTGGTCAATACGCTACATCTGCTGGGAACTCTGCTTCCGCTGCGCATCAATCTGAGGTAAACGCTGAGAACTATGCCACAGCATCCGCTAACTCTGCTCATTTGGCAGAACAGCAAGCAGACCGTGCGGAACGTGAGGCAGACAAGCTGGAAAATTACAATGGATTGGCTGGTGCAATTGATAAGGTAGATGGAACCAATGTGTACTGGAAAGGAAATATTCACGCTAACGGGCGCCTTTACATGACCACAAACGGTTTTGACTGTTGCCAGTATCAAc                <  1:351391/301‑1 (MQ=255)`  
                                                                                                                                                                                                                                                                                            `gCCAAGCGGTTAAAGGATACGGCTGGTCAATACGCTACATCTGCTGGGAACTCTGCTTCCGCTGCACATCAATCTGAGGTAAACGCTGAGAACTCTGCCACAGCATCCGCTAACTCTGCTCATTTGGCAGAACAGCAAGCAGACCGTGCGGAACGTGCGGCAGACAAGCTGGAAAATTACAATGGATTGGCTGGTGCAATTGATAAGGTAGATGGAACCAATGTGTACTGGAAAGGAAATATTCACGCTAACGGGCGCCTTTACATGACCACAAACGGTTTTGACTGTGGCCAGTATCaaa                >  1:594820/1‑300 (MQ=255)`  
                                                                                                                                                                                                                                                                                             `ccgaGCGGTTCAAGAATACGGCTGGTCAATACGCTACATCTGCTTGGAACTCTGCTTCCGCTGCGCATCAATCTGAGGTAAACGCTGAGAACTATGCCACAGCATCCGCTAACTATGCTCATTTGGCAGAACAGCAAGCAGACCGTGCGGAACGTGAGGCAGACAAGCTTGAAAATTACAATGGATTGGCTGGTGCAATTGATAAGGTAGATGGAACCAATGTGTACTGTAAAGGAAATATTCACGCTAACGGGCGCCTTTACATGACCACAAACGGTTTTGACTGTGGCCAGTATCAACa               <  1:142518/298‑1 (MQ=255)`  
                                                                                                                                                                                                                                                                                              `cgaGCGGTTCAAGAATACGGATGGTCAATACGCTACATCTGCTGGGAACTCTGCTTCCGCTGCGCATCAATCTGATGTAAACGCTGAGAACTCTGCCACAGCATCAGCTAACTCTGCTCATTTGGCAGAACAGCAAGCAGACCGTGCGAAACGTGAGGCAGACAAGCTGGAAAATTACAATGGATTGGCTGGTGCAATTGATAAGGTAGATGGAACCAATGTGTACTGGAAAGGAAATATTCACGCTAACGGGCGCCTTTACATGACCACAAACGGTTTTGACTGTGGCCAGTATCAACAg              <  1:253672/299‑1 (MQ=255)`  
                                                                                                                                                                                                                                                                                              `cAAGCGGTTCAAGAGTACGGCTGGTCAATACGCTACTTCTGCTTGGAACTCTGCTTCCGCTGCGCATCAATCTGAGGTAAACGCTGAGAACTATGCCACAGCATCCGCTAACTATGCTCATTTGGCAGAACAGCAAGCAGACCGTGCGGAACGTGAAGCAGACAAGCTGGAAAATTACAATGGATTGGCTGGTGCAATTGATAAGGTAGATGGAACCAATGTGTACTGGAAAGGAAATATTCACGCTAACGGGCGCCTTTACATGACCACAAACGGTTTTGACTGTGGCCAGTATCAACAg              <  1:309333/301‑1 (MQ=255)`  
                                                                                                                                                                                                                                                                                                    `gTTCAAGAATACGGCTGGTCAATACGCTACATCTGCTGGAAACTCTGCTTCCGCTGCGCATCAATCTGAGGTAAACGCTGAGAACTCTGCCACAGCATCCGCTAACTATGCTCATTTGGCAGAACAGCAAGCAGACCGTGCGGAACGTGAGGCAGACAAGCTGGAAAATTACAATGGATTGGCTGGTGCAATTGATAAGGTAGATGGAACCAATGTGTACTGGAAAGGAAATATTCACGCTAACGGGCGCCTTTACATGACCACAAACGGTTTTGACTGTGGCCAGTATCAACCGTTCttt        >  1:583029/1‑301 (MQ=255)`  
                                                                                                                                                                                                                                                                                                          `gAATACGGCTGGTCAATACGCTACATCTGCTGGGAACTCTGCTTCCGCTGCGCATCAATCTTAGGTAAACGCTGAGAACTCTGCCACAGCATCCGCTAACTATGCTCATTTGGCAGAACAGCAAGCAGACCGTGCGGAACGTGAGGCAGACAAGCTGGAAAATTACAATGGATTGGCTGGTGCAATTGATAAGGTAGATGGAACCAATGTGTACTGGAAAGGAAATATTCACGCTAACGGGCGCCTTTACATGACCACAAACGGTTTTGACTGTGGCCAGTATCAACCGTTCTTTggtggt  >  1:476112/1‑301 (MQ=255)`  

                                                                                                                                                                                                                                                                                                           `|`                                                                                                                                                                                                                                                                                                             
`ACCTCACTACGGATACTATCGGTGTCAATAACGATGGTCACTTGGATGCTCGTGGTCGTCGAATTGTGAACCTAGCGAACGCCGTGGATGACCGCGATGCTGTTCCGTTTGGTCAACTAAAGACCATGAACCAGAACTCATGGCAAGCACGTAATGAAGCCTTACAGTTCCGTAATGAGGCTGAGACTTTCAGAAACCAAGCGGAGGGCTTTAAGAACGAGTCCAGTACCAACGCTACGAACACAAAGCAGTGGCGCGATGAGACCAAGGGTTTCCGAGACGAAGCCAAGCGGTTCAAGAATACGGCTGGTCAATACGCTACATCTGCTGGGAACTCTGCTTCCGCTGCGCATCAATCTGAGGTAAACGCTGAGAACTCTGCCACAGCATCCGCTAACTCTGCTCATTTGGCAGAACAGCAAGCAGACCGTGCGGAACGTGAGGCAGACAAGCTGGAAAATTACAATGGATTGGCTGGTGCAATTGATAAGGTAGATGGAACCAATGTGTACTGGAAAGGAAATATTCACGCTAACGGGCGCCTTTACATGACCACAAACGGTTTTGACTGTGGCCAGTATCAACAGTTCTTTGGTGGT  >  NC_001604.1/34967‑35565`

  

Alignment Legend

Aligned base mismatch/match (shaded by quality score): `ATCG/ATCG` `< 3 ≤` `ATCG/ATCG` `< 11 ≤` `ATCG/ATCG` `< 26 ≤` `ATCG/ATCG` `< 36 ≤` `ATCG/ATCG` `< 38 ≤` `ATCG/ATCG`

Unaligned base: `atcg`    Masked matching base: `atcg`    Alignment gap: `‑`    Deleted base: `‑`
